# Access to the left intrahepatic bile duct using an endoscopic ultrasound-guided rendezvous technique

**DOI:** 10.1055/a-2218-2329

**Published:** 2024-01-09

**Authors:** Nobuhiko Hayashi, Toshiki Entani, Jun Matsuno, Ichiro Yasuda

**Affiliations:** 1 34823 Third Department of Internal Medicine, University of Toyama, Toyama, Japan

Appropriate preoperative biliary drainage in patients with hilar cholangiocarcinoma and severe jaundice reduces mortality after extensive hepatic resection. In this situation, endoscopic transpapillary drainage of the future remnant liver is generally performed as the first-line approach 1 . However, it is sometimes difficult to pass the guidewire into the target biliary branch because of stenosis and steep angulation of the bile duct.

A 70-year-old woman visited another hospital because of jaundice. A computed tomography scan suggested hilar cholangiocarcinoma ( Fig. 1 ). Endoscopic nasobiliary drainage (ENBD) of the right anterior branch was performed; however, her jaundice did not improve. The patient was therefore transferred to our hospital. Additional transpapillary biliary drainage of the left lobe was attempted; however, this failed because the guidewire could not be passed through the stenosed and steeply angulated left hepatic duct ( Fig. 2 ).

**Fig. 1:**
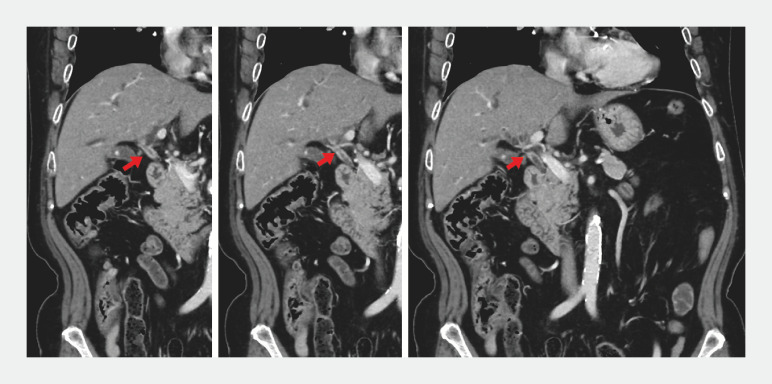
Contrast-enhanced computed tomography images showing a stricture and wall thickening (red arrow), with contrast enhancement of the hepatic hilar bile ducts.

**Fig. 2:**
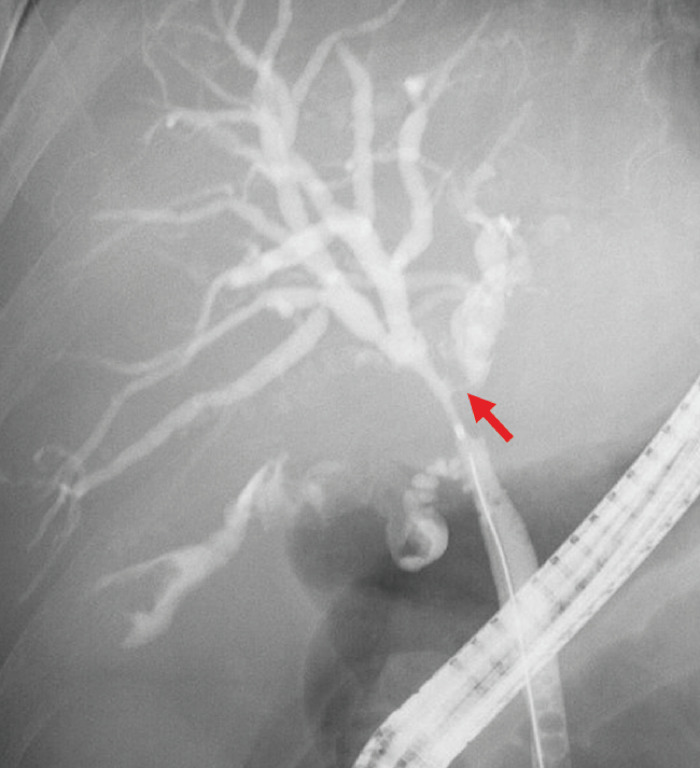
Fluoroscopic image during endoscopic retrograde cholangiography showing severe stenosis with steep angulation of the left hepatic duct (red arrow), such that a guidewire could not be passed through the stricture.

We therefore attempted an endoscopic ultrasound (EUS)-guided rendezvous approach. First, B3 was punctured by a 22G fine-needle aspiration (FNA) needle. We then attempted to advance a 0.018-inch guidewire beyond the stenosed left hepatic duct in an antegrade manner. The guidewire was successfully passed through the stricture and advanced to the duodenum. After the scope had been switched to a duodenoscope, the guidewire was grasped with forceps in the duodenum and was pulled into the scope channel. An endoscopic retrograde cholangiopancreatography (ERCP) catheter was then inserted into B3 over the guidewire. After removal of the guidewire, another guidewire was inserted in a transpapillary fashion into B3 through the catheter. A 5-Fr ENBD was inserted into B3, and an additional 5-Fr ENBD was inserted into the right posterior branch ( Video 1 ). Subsequently, the patient’s jaundice improved sufficiently, and an extended right hepatectomy was performed after right portal vein embolization.

An endoscopic ultrasound-guided rendezvous approach is successfully used in a patient with hilar bile duct cancer after failure to achieve selective access into the left bile duct by conventional endoscopic retrograde cholangiopancreatography.Video 1

In cases where selective access to the left intrahepatic bile duct fails while attempting endoscopic transpapillary biliary drainage, the EUS-guided rendezvous technique may be an effective salvage method.

Endoscopy_UCTN_Code_TTT_1AS_2AD

## Notes

Endoscopy E-Videos https://eref.thieme.de/e-videos
*E-Videos* is an open access online section of the journal *Endoscopy* , reporting on interesting cases and new techniques in gastroenterological endoscopy. All papers include a high-quality video and are published with a Creative Commons CC-BY license. Endoscopy E-Videos qualify for HINARI discounts and waivers and eligibility is automatically checked during the submission process. We grant 100% waivers to articles whose corresponding authors are based in Group A countries and 50% waivers to those who are based in Group B countries as classified by Research4Life (see: https://www.research4life.org/access/eligibility/ ). This section has its own submission website at https://mc.manuscriptcentral.com/e-videos .
